# Truncating mutations in *SPAST* patients are associated with a high rate of psychiatric comorbidities in hereditary spastic paraplegia

**DOI:** 10.1136/jnnp-2017-315796

**Published:** 2017-06-01

**Authors:** Viorica Chelban, Arianna Tucci, David S Lynch, James M Polke, Liana Santos, Hallgeir Jonvik, Stanislav Groppa, Nicholas W Wood, Henry Houlden

**Affiliations:** 1 Department of Molecular Neuroscience, UCL Institute of Neurology, London, UK; 2 National Hospital for Neurology and Neurosurgery, London, UK; 3 Department of Neurology and Neurosurgery, Institute of Emergency Medicine, Chisinau, Republic of Moldova; 4 Division of Pathology, Fondazione IRCCS Ca’ Granda Ospedale Maggiore Policlinico, Milano, Italy; 5 Department of Pathophysiology & Transplantation, Università degli Studi di Milano, Milano, Italy; 6 Neurogenetics Laboratory, UCL Institute of Neurology, The National Hospital for Neurology and Neurosurgery, London, UK

## Abstract

**Background:**

The hereditary spastic paraplegias (HSPs) are a rare and heterogeneous group of neurodegenerative disorders that are clinically characterised by progressive lower limb spasticity. They are classified as either ‘pure’ or ‘complex’ where spastic paraplegia is complicated with additional neurological features. Mutations in the spastin gene (*SPAST*) are the most common cause of HSP and typically present with a pure form.

**Methods:**

We assessed in detail the phenotypic and genetic spectrum of *SPAST*-related HSP focused on 118 patients carrying *SPAST* mutations.

**Results:**

This study, one of the largest cohorts of genetically confirmed spastin patients to date, contributes with the discovery of a significant number of novel *SPAST* mutations. Our data reveal a high rate of complex cases (25%), with psychiatric disorders among the most common comorbidity (10% of all *SPAST*patients). Further, we identify a genotype–phenotype correlation between patients carrying loss-of-function mutations in *SPAST* and the presence of psychiatric disorders.

## Introduction

The hereditary spastic paraplegias (HSPs) are a rare and heterogeneous group of neurodegenerative disorders characterised by slowly progressive lower limb spasticity^1^ with a prevalence that varies from 1.2 to 9.6 per 100 000.^2^ HSPs are classified as ‘pure’ when spastic paraplegia is the only clinical feature or ‘complex’ when is associated with other symptoms.^3^

Mutations in >70 distinct loci (SPG 1–72) and >50 genes have been identified in patients with HSPs.^4 5^ Mutations in the spastin gene (*SPAST*) are the most common cause of HSP, accounting for 45% of all autosomal-dominant forms.^6 7^ *SPAST* is an ATPase, belonging to the AAA family, involved in microtubule dynamics.^8 9^ All types of mutations have been described in *SPAST* with missense mutations clustered mainly in the AAA domain, while nonsense, splice-site mutations and insertions/deletions can be found in different locations throughout the gene.^10^

Clinically, *SPAST* is most often associated with a pure form of HSP. Complex phenotypes have been rarely described in *SPAST* and include seizures, intellectual disability and cerebellar ataxia.^11–13^ Neuropsychiatric comorbidities have only been reported in one study, where a higher-than-expected rate of psychosis was found in individuals with HSPs including SPAST;^14^ however, the association with spastin was uncertain. Depression is reported to be frequent (41%) but unrelated to disease severity.^15^ No clear correlation between type or location of the mutation and *SPAST* phenotype has been reported.

The clinical and genetic description, together with a detailed genotype–phenotype correlation in HSPs, is often limited due to small sample sizes of patients reported. We present here the results from one of the largest clinical and genetic cohorts of patients carrying *SPAST* mutations.

## Materials and methods

### Sample group

A totalof 118 patients from 104 families from the UK population referred as *SPAST* positive to the National Hospital for Neurology and Neurosurgery in London, a national referral centre for HSP.

### Genotype

Blood samples for DNA testing were collected with informed consent. Screening for point mutation in *SPAST* gene was undertaken by gene fluorescent DNA sequencing. Screening for whole-exon deletion and duplication was undertaken by Multiplex Ligation-dependent Probe Amplification analysis. For each reported mutation, we confirmed pathogenicity and novelty. For missense variants, prediction was based on residue conservation across different species and by looking at previously described mutations in the same residue. Frameshift and nonsense mutations as well as large deletions are predicted to result in the production of unstable messenger RNAs (mRNAs), truncated or absent proteins and were considered likely pathogenic. For splice-site mutation, in silico analysis software was used (Alamut) to determine whether the variant affects normal pre-mRNA splicing via disruption/creation of splice-site consensus sequences (http://www.interactive-biosoftware.com). The numbering of mutations in the text is consistent with GenBank (accession number NM_014946.3 for complementary DNA, NG_008730.1 for gene).

### Phenotype

Detailed phenotypic data were retrospectively collected through a series of standardised forms including patient history, neurological examination and instrumental evaluations for each patient carrying *SPAST* pathogenic variants.

Comprehensive phenotypic data were available for 84 cases. Onset before and including 20 years old was defined as early onset as a generally recognised standard.^3^ Pure phenotype was defined as HSP characterised by corticospinal tract syndrome (CTS), with sphincter involvement and vibration loss accepted as additional features. Complex phenotype was defined as cases with CTS, plus at least one other symptom that cannot otherwise be explained by associated conditions.

Disability score was defined as: 0, asymptomatic; 1, able to walk but difficult to run; 2, uses one stick and/or orthosis; 3, uses two sticks/walker; 4, unable to walk, uses wheelchair. The rate of disease progression was calculated by dividing the current disability score by the disease duration and presented in percentage per year.

Disease severity was assessed using the Spastic Paraplegia Rating Scale (SPRS).^16^ We had information available to complete the SPRS for 33 cases. Spasticity was assessed using the modified Ashworth Scale (AS) (0–4).^17^ Quantification of spasticity was possible in 59 cases. Muscle power was measured according to the Medical Research Council muscle scale (0–5).

Psychiatric comorbidities were formally diagnosed and followed up by the mental health team for all but one symptomatic case reported here. Non-symptomatic patients have not been routinely screened by a psychiatrist specialist.

### Statistical analysis

The distribution of severity score and disease progression rate by genotype was determined using a t-test. Univariate analysis of variance was performed for the correlation between age at onset (AAO) and genotype. A t-test was carried out to analyse mean AAO and gender and correlation between AAO and mode of inheritance (maternal/paternal). Kruskal-Wallis analysis of variance was used for analysing non-parametric values such as correlation between mutation type/functional domain/AAO/gender and disease progression, disability score, AS and SPRS. p≤0.05 was the significance level used for statistical analysis. We used the SPSS (version 23.0) software for statistical analysis.

## Results

### Genetic spectrum

**Figure 1:**
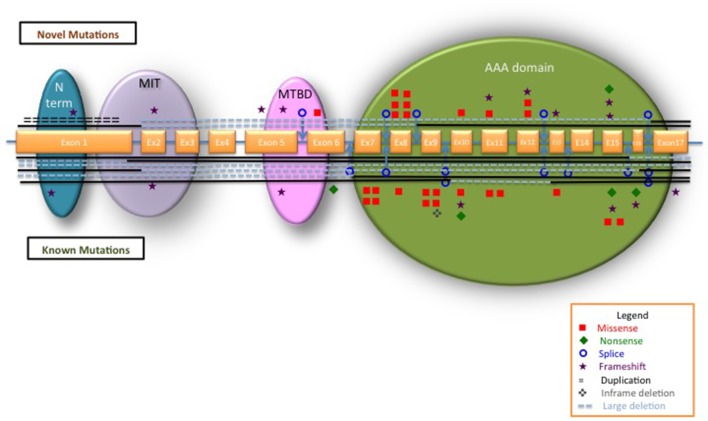
Schematic representation of *SPAST* with all the mutations identified in our study. The exons are represented approximately to scale. A blue line between exons represents the introns. The *SPAST* functional domains are indicated: blue, N-terminal sequence; purple, microtubule interacting and trafficking domain (MIT); pink, microtubule-binding domain (MTBD); green, AAA (ATPase associated with various cellular activities). Novel mutations are plotted on top of the gene; known mutations are below the gene. Types of different mutations are represented as per legend. Interrupted lines show the exons involved in large deletions.

We identified 118 cases from 104 families carrying *SPAST* variants. A total of 72 unique pathogenic variants were identified, of which 40.3% were novel (table 1) and 59.7% previously described (see online supplementary table e1). Ten cases carried variants of which the causal proof to HSP could not be established, including the previously described HSP phenotypic modifier p.Ser44Leu; these were removed from further analysis (see online supplementary table e2). The polymorphism p.Ser44Leu was found in five cases, three of which had a second *SPAST* pathogenic variant (alleles frequency 5/216, 0.2%) in keeping with the Minor allele frequency of 0.9% reported in European/Americans in Exome Variant Server. The vast majority of mutations were unique to one kindred (82%) and only 13 were recurrent. The most frequent mutation is the p.Arg460Cys identified in five unrelated families (5.3%). All types of mutations were identified. Missense mutations represented 39%. Loss-of-function mutations represented 61% including framshift 22%, splicing 17%, nonsense 7%, inframe deletions 3%, duplications 1% and large whole-exon deletions 11%. While loss-of-function mutations are scattered throughout *SPAST*, all missense mutations are located in the AAA domain, with only one exception (the p.Ile328Lys) (figure 1).

10.1136/jnnp-2017-315796.supp1 Supplementary Table 1

**Table 1:** Description of all likely pathogenic novel mutations identified in this study

Family number	cDNA change	Amino acid change	E/I	Conclusion	Evidence
7	c.282_322del41	p.Ala96fs	E 1	Likely pathogenic	Frameshift
9	c.474delA	p.Gly159Glufs*2	E 2	Likely pathogenic	Frameshift
97	c.463_465del	p.155_155del	E2	Likely pathogenic	Frameshift
3	c.696insC	p.Arg235Lysfs*9	E 5	Likely pathogenic	Frameshift
12	c.831_835del5	p.Val278Thrfs*11	E 5	Likely pathogenic	Frameshift
15	c.871-2A>G	p.?	I 5	Likely pathogenic	Affects essential splice site. In silico splicing tools predict an effect on splicing
16	c.983T>A	p.IIe328Lys	E 6	Likely pathogenic	Novel missense mutation at highly conserved amino acid. SIFT and PolyPhen predict to be pathogenic
24	c.1102T>G	p.Phe368Val	E 8	Likely pathogenic	Novel missense mutation at highly conserved amino acid. SIFT and PolyPhen predict to be pathogenic
25	c.1104C>A	p.Phe368Leu	E 8	Likely pathogenic	Novel missense mutation at highly conserved amino acid. Different substitution at same amino acid previously reported (HGMD CM114203)
26	c.1156A>T	p.Asn386Tyr	E 8	Likely pathogenic	Novel missense mutation at highly conserved amino acid
28	c.1115G>C	Arg372Thr	E 8	Likely pathogenic	Novel missense mutation at highly conserved amino acid. Different substitution at same amino acid previously reported (HGMD CD021854, HGMD CI090392)
29	c.1130G>C	p.Gly377Glu	E 8	Likely pathogenic	Novel missense mutation at highly conserved amino acid. Different substitution at same amino acid previously reported (HGMD CM114634)
32	c.1169T>C	p.Met390Thr	E 8	Likely pathogenic	Novel missense mutation at highly conserved amino acid
33	c.1174–1G>A	p.?	I 8	Likely pathogenic	Affects essential splice site. In silico splicing tools predict an effect on splicing.
44	c.1253A>C	p.Glu418Ala	E 10	Likely pathogenic	Novel missense mutation at highly conserved amino acid
55	c.1406delT	p.Phe469Leufs*14	E 11	Likely pathogenic	Frameshift
56	c.1408G>T	p.Asp470Tyr	E 11	Pathogenic	Novel missense mutation at highly conserved amino acid. SIFT and PolyPhen predict to be pathogenic. Segregated in other family members
57	c.1442_1443insA	p.Val482Cysfs*6	E 12	Likely pathogenic	Frameshift
58	c.1453G>A	p.Ala485Thr	E 12	Likely pathogenic	Novel missense mutation at highly conserved amino acid
59	c.1493+2_1493+5delTAGG	p.?	I 12	Likely pathogenic	In silico splicing tools predict donor splice site abolished
61	c.1493G>T	p.Arg498Met	E 12	Likely pathogenic	Novel missense mutation at highly conserved amino acid. Different substitution at same amino acid previously reported (HGMD CM063165)
63	c.1535_1536+1delAGG	p.Glu512Aspfs*7	E 13	Likely pathogenic	Frameshift
66	c.1635_1636insAA	p.Gly546Lysfs*5	E 15	Likely pathogenic	Frameshift
68	c.1636G>T	p.Gly546X	E 15	Likely pathogenic	Frameshift
70	c.1664delA	p.Asp555Valfs*10	E 15	Likely pathogenic	Frameshift
84	c.1729–20T>G	p.?	I 16	Likely pathogenic	In silico splicing tools predict cryptic splice site activated
88	Duplication of exon 1	p.?	E 1	Pathogenic	Disruption leading to severe protein alteration
96	Deletion of exons 2–9	p.?	–	Pathogenic	Disruption leading to severe protein alteration
92	Deletion of exons 1–16	p.?	–	Pathogenic	Disruption leading to severe protein alteration

cDNA, complementary DNA; E, exon; I, intron.

All cases included in this study had a single heterozygous mutation except case 62, which carries two heterozygous missense mutations (p.Arg372Thr, p.Asn579His), and case 3, which was found to carry two *in-cis* mutations (p.Arg364Met, p.Pro365Ser). In case 62, we did not have DNA from any other family members and we were unable to establish if the two mutations were inherited *in-cis* or *in-trans*, but p.Asn579His has previously been reported as pathogenic both in association with a second mutation^18^ and in isolation.^19^ One case (37) presenting with two concurrent autosomal-dominant mutations (*SPAST* and *OPMD*) was described in details here^20^ and was removed from further phenotype analysis.

### Clinical features

**Figure 2:**
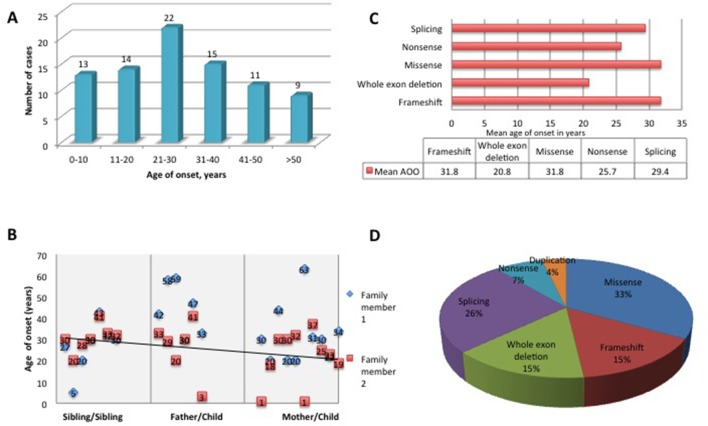
Mutation spectrum in *SPAST*-related hereditary spastic paraplegia (HSP). (A) Age at  onset (AAO) within families. (B) Correlation between the AAO within families with a regression line. The first part of the graph shows the AAO between siblings, the middle part of the graph shows AAO when the disease is inherited from the father and the last part of the graph shows the AAO when the disease is inherited from the mother. (C) AAO by mutation type. (D) Frequency of different mutation types in early-onset spastin HSP cases.

The mean AAO was 29.2±16.2 years with a range from birth to 63 years (figure 2A), with high variability between and within the families. Within families, a significantly earlier onset was observed in the offspring of affected parents (p=0.02) (figure 2B); however, we attribute this to recall and ascertain bias. Early onset, defined as onset before 20 years of age, was identified in 32.1%. There was no statically significant correlation between AAO and mutation type (figure 2C). However, it was noted that missense mutations were responsible for 33% of early-onset HSP and 46% of cases when AAO was ≤10 years (figure 2D). Onset before 10 years was identified in 14 patients (16%).

**Figure 3:**
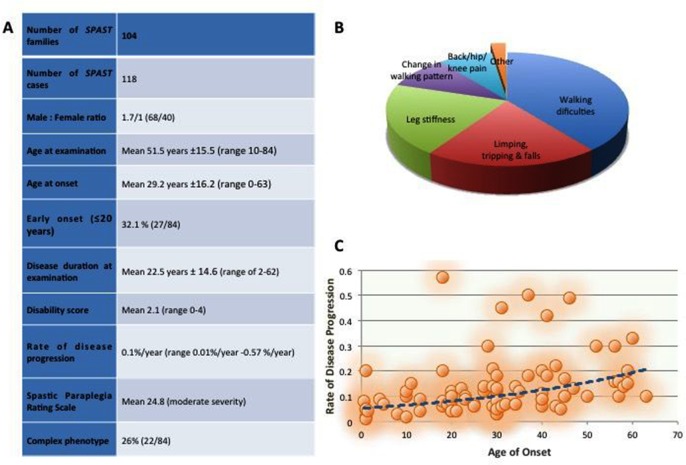
Clinical spectrum of *SPAST*-related hereditary spastic paraplegia. (A) Demographics of the *SPAST* cohort. (B) Symptoms of onset in *SPAST* patients. (C) Correlation between rate of disease progression and age at onset (AAO). AAO >41 was associated with a higher rate of progression (p=0.01).

The summary demographic data are presented in figure 3A. The most common symptom at onset was related to lower limbs spasticity in the vast majority of cases. It included walking difficulty (38%), leg stiffness (23%), limping and tripping (18%), tip-toe walking (8%) and back pain (6%) (figure 3B). One case initially reported neurogenic bladder (case 30) and another presented with delayed walking and learning difficulties (case 52 carried a whole-gene deletion).

Examination of the lower limbs was suggestive of a pyramidal syndrome with spasticity being more marked than weakness. Spasticity measured with AS revealed an average spasticity score of 1.8, while muscle power measured with Medical Research Council muscle scale revealed an average of 4/5 in a pyramidal pattern. Lower limbs sensory loss was present in 30% of patients and consisted mainly of decreased vibration sense.

Disease severity was measured with the SPRS, which revealed a mean score of 24.8 (of max 52), consistent with moderate severity. While there was no significant difference between SPRS and mutation type, a trend was observed between SPRS and age at onset (AAO): a higher SPRS score was observed in the late-onset group (mean SPRS score 26.2) compared with the early-onset (0–20 years AAO) (mean SPRS=21.5).

The mean disability score was 2.1. There is no significant difference between disability score and type of mutation. The mean rate of disease progression was 0.12%/year (SD ±0.12%) ranging from 0.01%/year to 0.57%/year, confirming a slow, but progressive course of the disease. By correlating disease progression with AAO, it was observed that later AAO (>40 years) is associated with a higher progression rate of the disease (p=0.01) (figure 3C).

#### Pure versus complex phenotype

Pure phenotype was present in 74% of cases consisting of slowly progressive lower limb spasticity and weakness. Of note, neurogenic bladder was reported in 58% of patients while neurogenic bowel symptoms were present in 26% of all patients.

Complex phenotype was identified in 26% of patients and presented as CTS plus cerebellar syndrome, peripheral neuropathy, learning disability, memory problems and psychiatric symptoms (table 2). Interestingly, 10.7% of patients (nine cases from seven different families) presented with an associated psychiatric illnesses. Three cases had autism spectrum disorders; three cases had a diagnosis of severe depression and single cases each had borderline personality disorder, hypomania and schizoaffective disorder, respectively. Of note, eight out of nine patients affected by psychiatric comorbidities carry loss-of-function mutations most of which located in the AAA domain. Strikingly, one mutation leading to premature stop codon (c.1635_1636insAA, p.Gly546Lysfs*5) was identified in four cases from three families. This is one of the few recurrent mutations in our cohort (frequency of 4.7%) and is fully penetrant for psychiatric comorbidities.

**Table 2:** Description of all complex *SPAST* cases

Case number	cDNA change	Mutation type	CTS	PN	Psychiatric comorbidity	Other
52	Deletion of exons 1–17	Whole coding *SPAST* sequence deletion	+	–	Autism spectrum disorder	–
75	c.1408G>T	Missense	+	SM PN	Autism spectrum disorder	Memory impairment, dysphagia, seizures
43	c.1635_1636insAA	Frameshift	+	–	Asperger	–
59	c.1805_1808dup AAGC	Frameshift	+	M PN	Severe depression under mental health team	Paraspinal schwannoma
94	c.1684C>T	Nonsense	+	–	Severe depression	Congenital torticollis, pes cavus
87	c.1635_1636insAA	Frameshift	+	–	Severe depression, gender identity disorder	–
54	c.1635_1636insAA	Frameshift	+	–	Schizoaffective disorder	–
7	c.1635_1636insAA	Frameshift	+	–	Abnormal behaviour, not formally diagnosed by mental health team yet	–
53	c.1174–1G>A	Splicing	+	–	Hypomania	–
82	Duplication of exon 1	Duplication	+	SM PN	–	Mild learning disability
41	c.1082C>T	Missense	+	–	–	Memory impairment, dysarthria, neurofibromatosis type 1
20	c.1728+2T>C	Splicing	+	–	–	Learning disability, seizures, periodic movements of sleep
21	c.1728+2T>C	Splicing	+	–	**–**	Memory impairment
26	c.1728+2T>C	Splicing	+	SM PN	–	–
58	c.1004+2T>A	Splicing	+	Axonal neuropathy on nerve biopsy	–	–
19	c.1728+1G>T	Splicing	+	M PN	–	–
66	c.1676insG	Frameshift	+	SM PN	–	–
80	c.1684C>T	Nonsense	+	M PN	–	–
83	Duplication of exon 1	Duplication	+	S PN	–	Seizures
117	Deletion of exons 10–12	Large deletion	+	–	–	Seizures
60	c.1378C>T	Missense	+	–	–	Ataxia, broken pursuit

+, present; –, absent; cDNA, complementary DNA; CTS, corticospinal tract syndrome; M, motor; PN, peripheral neuropathy; S, sensory.

Assessment of cognitive function revealed two cases of mild learning disability (cases 82 and 20) and three cases of memory impairment (case 75 is 46 years of age, case 41 is 62 years of age and case 21 is 77 years of age). One case underwent formal neuropsychology assessment, which revealed cognitive impairment affecting frontal and temporal functions. All three mutations associated with memory impairment in our cohort are missense variants located in the highly conserved AAA domain.

Nerve conduction studies were conducted in 34 patients (see online supplementary table e3) and identified axonal peripheral polyneuropathy in 23.5% with chronic denervation on Electromyography in 16% of cases. One case (80) showed slow motor conduction velocities approaching the demyelinating range. However, a selective bias cannot be excluded. Brain MRI was available in 45 cases and was normal in 85%. In the remaining 16%, 9% had mild-to-moderate thinning of the corpus callosum; one case had mild cerebellar atrophy (case 113), one had cysts in the posterior fossa (case 69) and one asymmetrical volume loss in the right hemisphere and midbrain (case 41) (see online supplementary figure e1). MRI of the spinal cord was performed in 10 patients with 8 of them reported as normal cord or degenerative changes only. Only two cases were reported with thinning of the spinal cord. There was no correlation between MRI changes and complex phenotypes.

Other neurological comorbidities in association with HSP identified in our cohort were single cases of confirmed secondary progressive Multiple Sclerosis, congenital torticollis, neurofibromatosis, schwannoma and cluster headache.

## Discussion

In the present cohort, we identified 72 unique pathogenic *SPAST* mutations in 108 cases from 94 British families, confirming previous results that recurrent mutations in *SPAST* are not common.^21^ All types of variants were identified, almost half of the patients presenting missense mutations. Our cohort confirms^22^ that the majority of missense mutations are found in the AAA domain confirming the importance of AAA cassette for *SPAST* function.^10 23^ This was linked with a possible dominant negative effect of these missense mutations on the gene function.^9^ In our study, when p.Ser44Leu phenotypic modifier was associated with a second proven causative mutation (case 44), it produced an early AAO (11 years) with a complex phenotype with a disability score of 4/4.

With >30% of patients presenting an early onset, including 16% before the age of 10, this study and recent research^22^ contradict the previous suggestion that spastin causes almost exclusively a late-onset HSP. Half of these cases were caused by highly disruptive, structural variation mutations. As *SPAST*-related HSP is considered an adult onset disease, these cases present a big challenge to differentiate with SPG3A (a predominantly pure HSP, where 80% of cases present before the first decade of life) and only the genetic testing can provide the definitive diagnosis.

Most importantly, we report over a quarter of our *SPAST* cases presenting a complex HSP phenotype, with >10% presenting with an associated psychiatric diagnosis. Psychiatric manifestations in HSP were previously described as very rare and only in single reports^11^ of unipolar depression, migraine or euphoria.^24 25^ Depression was the most common mood disorder associated with HSP in a previous study described as mild to moderate and only single cases of severe depression have been reported.^15^ We present three cases (3.5%) of severe depression requiring treatment under the mental health team and single cases of borderline personality disorder, hypomania and schizoaffective disorder. Manic and affective disorders are very rare in HSP.^26 27^ There was an association between *SPAST* and psychosis in an Irish cohort;^14^ but with a small number of cases (48) and psychotic disorders being fairly common in general population (1% in schizophrenia), these results could be associated to chance. Nevertheless, both our study and the Irish one show an increased rate of psychiatric illnesses in spastin patients and further prospective studies using detailed psychiatric assessments are necessary to establish a correlation. More surprisingly, we found that 3.5% of cases in our cohort had an autistic spectrum disorder; all diagnosed by psychiatrists according to internationally approved diagnostic criteria. To our knowledge, there are no reports of an association between autism and spastin or HSP. It is estimated that about 1% of the UK population suffers from an autism spectrum disorder^28^ well below the rate associated with spastin in our series. Interestingly, we found one causal mutation associated with psychiatric illness and *SPAST*-related HSP that had an unusual high frequency of 3.5% in our cohort. The novel c.1635_1636insAA frameshift was identified in four cases from three families. Of note, the vast majority of the mutations associated with psychiatric manifestations are loss-of-function mutations affecting the AAA functional domain.

So far, there is no strong evidence from Genome Wide Association Study or Copy Number Variants studies for autism loci found near or comprising *SPAST*.^29 30^ A recent study showed that inherited Single Nucleotide Variants that produce truncated proteins are more frequent in autistic probands and are in genes that are intolerant for functional variations.^31^ Furthermore, they found one case with autism and truncating Single Nucleotide Variants in the exon 5 of *SPAST*.^31^

The mechanism behind the psychiatric manifestations in HSP remains unclear but it has been associated with thalamic hypoperfusion,^32^ thin corpus callosum or white matter abnormalities.^33^ Cysts in the posterior fossa have been previously reported in *SPAST* cases.^34^ Nevertheless, these data should be cautiously interpreted as some of these changes could reflect developmental rather than neurodegenerative changes and studies with long-term follow-up will be useful in distinguishing between the two, such as serial MRIs in assessing thinning of the corpus callosum. Also, we had formal neuropsychiatry assessment for the symptomatic cases only, limiting our ability to comment on the extent of psychiatric comorbidities and spastin. However, our study warrants further investigations and future studies would have to systematically screen spastin patients with a uniform evaluation in order to validate the findings.

In our cohort, we report 4.7% of patients with genetically diagnosed spastin mutation and learning disabilities and 3.5% of seizures. Interestingly, all three cases with seizures reported in our study were associated with cognitive deficit. Very few reports describe this association and *SPAST* .^35^

We also identified memory impairment in 3.5% of cases. We are limited in our ability to further describe them due to the lack of any histopathological diagnosis in these cases. The association between HSP and dementia could be explained by coincidence given the small numbers and no extensive neuropathology studies in all reported series. A small number of neuropsychology reports have associated spastin with subcortical dementia,^12 36^ correlating with age, but not with the degree of spasticity. Genotypes of the reported families with cognitive decline and spastin included missense mutations (Ala139Gly)^37^ as well as large deletion of exon 17.^38^ One *SPAST*-affected family has been previously reported in the literature as having a very unusual tau pathology on postmortem brain biopsy.^37^ As spastin does not limit lifespan, caution is required when interpreting postmortem results as some changes can be age related or due to concurrent diseases.

Correlation between mutation type and phenotype has not been reported so far. We show that although not statistically significant, there is a trend of earlier AAO associated with large deletions, splicing and missense mutations. We confirm that a later AAO is associated with a higher rate of disease progression as previously reported^3 10^ and a higher degree of spasticity. There was no other genotype–phenotype correlation.

Our study confirms previous reports that polyneuropathy associated with *SPAST* is predominantly axonal motor neuropathy with only mild slowing of conduction velocities.^39 40^

This study, one of the largest cohorts of genetically confirmed spastin patients to date, contributes with the discovery of a significant number of novel *SPAST* mutations and demonstrates the high genotype–phenotype variability in the disease. Most importantly, it highlights the high prevalence of psychiatric comorbidities in patients with HSP caused by a *SPAST* mutation. Of note, the previously under-recognised association with autism and psychiatric illness needs to be confirmed in other cohorts and genes to determine if this is a specific effect of *SPAST* mutations.

10.1136/jnnp-2017-315796.supp2 Supplementary Figure 1
