# Supplementary material for: Truncating mutations in SPAST patients are associated with a high rate of psychiatric comorbidities in hereditary spastic paraplegia
Source: J Neurol Neurosurg Psychiatry. 2017 Jun 1;88(8):681–7. doi: 10.1136/jnnp-2017-315796 (PMC5537546; doi:10.1136/jnnp-2017-315796)
Supplement: Supplementary Table 1 [file jnnp-2017-315796supp001.pdf]

Supplementary material

Table e1. DESCRIPTION OF ALL KNOWN SPASTIN MUTATIONS INCLUDED IN THE STUDY

Family number	Case number	cDNA sequence	Amino acid change	Location	Functional Domain	Heterozygous/Homozygous	Consequence of mutation	Type of mutation	Accession number (AN)	MAF in Exac
1	48	c.85dupC	p.Leu29fs*18	Exon 1	N-term	Heterozygous	Frameshift	small indel	CI063729	NA
2	57	c.68G>A	p.Arg23Lys	Exon 1	N-term	Heterozygous	Missense	SNV	rs558882317	MAF 0.0008/4
4	88	c.131C>T	p.Ser44Leu	Exon 1	N-term	Heterozygous	Missense	SNV	rs121908515	MAF 0.005415
5	95	c.131C>T	p.Ser44Leu	Exon 1	N-term	Heterozygous	Missense	SNV	rs121908515	MAF 0.005415
6	56	c.131C>T	p.Ser44Leu	Exon 1	N-term	Heterozygous	Missense	SNV	rs121908515	MAF 0.005415
10	92	c.484G>A	p.Val162Ile	Exon 2	MIT	Heterozygous	Missense	SNV	rs141944844	MAF 0.001879
11	30<br>84	c.486dupT	p.Ile163Tyrfs*7	Exon 2	MIT	Heterozygous	Frameshift	small indel	CI063728	NA
13	93	c.843_846dupATCT	p.Gly283Ilefs*9	Exon 5	MTBD	Heterozygous	Frameshift	small indel	CI076999	NA
14	111	c.843_846dupATCT	p.Gly283Ilefs*9	Exon 5	MTBD	Heterozygous	Frameshift	small indel	CI076999	NA
17	16	c.983_984dupTA	p.Met329*	Exon 6	Outside domain	Heterozygous	Nonsense	small indel	CI090391	NA
18	58	c.1004+2T>A	/	Intron 6	AAA	Heterozygous	Splicing	SNV	CS000465	NA

Family number	Case number	cDNA sequence	Amino acid change	Location	Functional Domain	Heterozygous/Homozygous	Consequence of mutation	Type of mutation	Accession number (AN)	MAF in Exac
19	37	c.1082C>T	p.Pro361Leu	Exon 7	AAA	Heterozygous	Missense	SNV	CM042482	NA
	41	c.1082C>T	p.Pro361Leu	Exon 7	AAA	Heterozygous	Missense	SNV	CM042482	NA
20	3	c.1091G>T; 1093C>T	p.Arg364Met;p.Pro365Ser	Exon 7	AAA	*in-cis* mutation	Missense	SNV	CM11420; CM103636	NA; NA
21	33	c.1096G>A	p.Glu366Lys	Exon 7	AAA	Heterozygous	Missense	SNV	Published but no AN	NA
22	29	c.1098+1G>A	/	Exon 7	AAA	Heterozygous	Splicing	SNV	CS063390 or CS000466	NA
30	32	c.1157A>G	p.Asn386Ser	Exon 8	AAA	Heterozygous	Missense	SNV	CM044068 - rs121908514	NA
35	23	c.1196C>T	p.Ser399Leu	Exon 9	AAA	Heterozygous	Missense	SNV	CM022250	NA
36	81	c.1209_1211delCTT	p.404del	Exon 9	AAA	Heterozygous	Inframe deletion	small indel	CD052506	NA
37	70	c.1209C>G	p.Phe403Leu	Exon 9	AAA	Heterozygous	Missense	SNV	CM063175	NA
	45									
	109									
38	67	c.1216A>G	p.IIe406Val	Exon 9	AAA	Heterozygous	Missense	SNV	CM060485	NA
39	69	c.1216A>G	p.IIe406Val	Exon 9	AAA	Heterozygous	Missense	SNV	CM060485	NA
40	15	c.1217T>G	p.Ile406Arg	Exon 9	AAA	Heterozygous	Missense	SNV	CM060485	NA
41	49	c.1245+1G>A	/	Intron 9	AAA	Heterozygous	Splicing	SNV	CS011845	NA
	39									

Family number	Case number	cDNA sequence	Amino acid change	Location	Functional Domain	Heterozygous/Homozygous	Consequence of mutation	Type of mutation	Accession number (AN)	MAF in Exac
42	36	c.1245+1G>A	/	Intron 9	AAA	Heterozygous	Splicing	SNV	CS011845	NA
43	42	c.1245+5G>A	/	Intron 9	AAA	Heterozygous	Splicing	SNV	CS011845	NA
45	9	c.1285delG	p.Val429fs	Exon 10	AAA	Heterozygous	Frameshift	small indel	CD114205	NA
46	76	c.1291C>T	p.Arg431*	Exon 10	AAA	Heterozygous	Nonsense	SNV	CM000437	NA
47	97	c.1291C>T	p.Arg431*	Exon 10	AAA	Heterozygous	Nonsense	SNV	CM000437	NA
48	103	c.1307C>T	p.Ser436Phe	Exon 10	AAA	Heterozygous	Missense	SNV	CM00477	NA
49	5	c.1350A>T	p.Arg450Ser	Exon 11	AAA	Heterozygous	Missense	SNV	CM114204	NA
50	107	c.1378C>T	p.Arg460Cys	Exon 11	AAA	Heterozygous	Missense	SNV	CM042786	NA
51	60	c.1378C>T	p.Arg460Cys	Exon 11	AAA	Heterozygous	Missense	SNV	CM042786	NA
52	89	c.1378C>T	p.Arg460Cys	Exon 11	AAA	Heterozygous	Missense	SNV	CM042786	NA
53	112	c.1378C>T	p.Arg460Cys	Exon 11	AAA	Heterozygous	Missense	SNV	CM042786	NA
54	113	c.1378C>T	p.Arg460Cys	Exon 11	AAA	Heterozygous	Missense	SNV	CM042786	NA
60	10	c.1493+18G>T	/	Intron 12	AAA	Heterozygous	Splicing	SNV	rs189961829	MAF 0.003066
62	101	c.1508G>T	p.Arg503Leu	Exon 13	AAA	Heterozygous	Missense	SNV	CM030281	NA
64	55	c.1536+2T>G	/	Intron 13	AAA	Heterozygous	Splicing	SNV	CS021767	NA
69	85	c.1649C>T	p.Thr550Ile	Exon 15	AAA	Heterozygous	Missense	SNV	CM065472	NA
71	66	c.1676insG	p.Pro560Serfs*17	Exon 15	AAA	Heterozygous	Frameshift	small indel	CI114206	NA
72	72	c.1684C>T	p.Arg562*	Exon 15	AAA	Heterozygous	Nonsense	SNV	rs1219085	NA

**Family number**	**Case number**	**cDNA sequence**	**Amino acid change**	**Location**	**Functional Domain**	**Heterozygous/Homozygous**	**Consequence of mutation**	**Type of mutation**	**Accession number (AN)**	**MAF in Exac**
	77<br>80								18, CM000441	
73	12	c.1684C>T	p.Arg562*	Exon 15	AAA	Heterozygous	Nonsense	SNV	rs121908518, CM000441	NA
74	94	c.1684C>T	p.Arg562*	Exon 15	AAA	Heterozygous	Nonsense	SNV	rs121908518, CM000441	NA
75	106	c.1684C>T	p.Arg562*	Exon 15	AAA	Heterozygous	Nonsense	SNV	rs121908518, CM000441	NA
76	64	c.1685G>A	p.Arg562Gln	Exon 15	AAA	Heterozygous	Missense	SNV	CM022254	NA
77	91	c.1687+1G>T	/	Intron 15	AAA	Heterozygous	Splicing	SNV	CS107591	NA
78	99	c.1702C>T	p.Gln568*	Exon 16	AAA	Heterozygous	Nonsense	SNV	CM090418	NA
79	110	c.1720delG	p.Ala574Profs*4	Exon 16	AAA	Heterozygous	Frameshift	small indel	CD030353	NA
80	2	c.1728+1G>A	/	Intron 16	AAA	Heterozygous	Splicing	SNV	CS000472	NA
81	68<br>19	c.1728+1G>T	/	Intron 16	AAA	Heterozygous	Splicing	SNV	CS002468	NA
82	26	c.1728+2T>C	/	Intron 16	AAA	Heterozygous	Splicing	SNV	CS004671	NA

Family number	Case number	cDNA sequence	Amino acid change	Location	Functional Domain	Heterozygous/Homozygous	Consequence of mutation	Type of mutation	Accession number (AN)	MAF in Exac
83	21	c.1728+2T >C	/	Intron 16	AAA	Heterozygous	Splicing	SNV	CS004671	NA
87	20	c.1728+2T >C	/	Intron 16	AAA	Heterozygous	Splicing	SNV	CS004671	NA
28	62	1735A>C	p.Asn579His	Exon 17	AAA	Heterozygous	Missense	SNV	CM054863	NA
86	59<br>102	c.1805_1808dup AAGC	p.Tyr604Serfs*28	Exon 17	Outside domain	Heterozygous	Frameshift	small indel	CI114207	NA
89	65	Deletion of exon 1	-	-	N-TERM/MIT	Heterozygous	Whole exon deletion	whole exon CNV	CG072715	NA
90	47	Deletion of exon 1	-	-	N-TERM/MIT	Heterozygous	Whole exon deletion	whole exon CNV	CG072715	NA
91	105	Deletion of exon1-7	-	-	ALL	Heterozygous	Whole exon deletion	whole exon CNV	CG066493	NA
93	52	Deletion of exons 1-17	-	-	ALL	Heterozygous	Whole exon deletion	whole exon CNV	CG072716	NA
94	22	Deletion of exons 2-16	-	-	ALL	Heterozygous	Whole exon deletion	whole exon CNV	CG073890	NA

Family number	Case number	cDNA sequence	Amino acid change	Location	Functional Domain	Heterozygous/Homozygous	Consequence of mutation	Type of mutation	Accession number (AN)	MAF in Exac
95	25	Deletion of exons 16-17	-	-	ALL	Heterozygous	Whole exon deletion	whole exon CNV	CG066497	NA
	28	Deletion of exons 16-17	-	-	ALL	Heterozygous	Whole exon deletion	whole exon CNV	CG066497	NA
96	24	131G>T, deletion of exons 2-9	p.[Ser44Leu(+) deletion]	Exon 1, whole exon 2-9	ALL	Heterozygous	Missense, Whole exon deletion	SNV and whole exon CNV	rs121908515	MAF 0.005415
97	44	131G>T, 463_465del	p.Ser44Leu, p.155_155del	Exon 1, exon 2	N-TERM/MIT	Heterozygous	Missense, Inframe deletion	SNV, small indel	rs121908515	MAF 0.005415

Table S1. DESCRIPTION OF ALL KNOWN MUTATIONS INCLUDED IN THE STUDY.

Table e2. Variants of unknown significance

Family #	Case #	c.DNA change	Amino acid change	E/I	Functional Domain	Consequence of mutation	Effect
2	57	c.68G>A	p.Arg23Lys	E 1	N-term	Missense	Unknown significance
8	100	c.315G	p.Pro105Pro	E 1	Outside	Synonymous/	Unknown

		>A			domain	silent	significance
4,5,6	88,95 ,56	c. 131C>T	p.Ser44Leu	E1	N-term	Missense	Intragenic modifier, Unkown significance
103, 10	119, 92	c.484G >A	p.Val162Ile	E 2	MIT	Missense	Unkown significance
27	27	c.1107A >G	p.Thr369Thr	E 8	AAA	Synonymous/ silent	Unknown significance
23	79	c.1099-35A>G	/	I 7	AAA	Splicing	Unknown significance
31	38	c.1155G >A	p.Gly385Gly	E 8	AAA	Synonymous/ silent	Unknown significance

Table S2. Variants of unknown significance. #-Number; /-splicing; E-exon; I-Intron.

Table e3. NCS RESULTS IN *SPAST* PATIENTS

Case #	MOTOR NCS										SENSORY NCS					
	Median nerve			Ulnar nerve			Tibial nerve			MEP	Median nerve		Ulnar nerve		Sural nerve	
	DL <4 ms	CMAP amp >8mV	MCV >50 m/s	DL <3.5 ms	CMAP amp >8 mV	MCV >51 m/s	DL <5.5 ms	CMAP amp >7.5 mV	MCV >40 m/s	LL <17 ms	SNAP amp >10mV	SCV >49 m/s	SNAP amp >13mV	SCV >52 m/s	SNAP amp >9mV	SCV >40 m/s
**13**	4	7.7	51	2.3	9.9	53	4.4	4.3	44	na	5	48	5	48	5	45
**53**	4.4	7.3	52	2.6	12.5	59	4.9	8.7	53	na	20	54	10	49	18	51
**71**	3.7	11.1	53	na	na	na	6.5	0.6	na	na	9	54	5	50	14	48
**3**	4.8	11.6	48	3.4	10.4	51	5.9	4.8	42	na	8	41	5	40	7	50
**11**	3.9	7.2	49	2.3	9	59	na	na	na	na	8	36	6	63	na	na

Case #	MOTOR NCS										SENSORY NCS					
	Median nerve			Ulnar nerve			Tibial nerve			MEP	Median nerve		Ulnar nerve		Sural nerve	
	DL <4 ms	CMAP amp >8mV	MCV >50 m/s	DL <3.5 ms	CMAP amp >8 mV	MCV >51 m/s	DL <5.5 ms	CMAP amp >7.5 mV	MCV >40 m/s	LL <17 ms	SNAP amp >10mV	SCV >49 m/s	SNAP amp >13mV	SCV >52 m/s	SNAP amp >9mV	SCV >40 m/s
**18**	na	na	na	3.1	9.4	50	5.4	7.9	44	na	26	59	7	61	14	45
**26**	8.5	6	53	na	na	na	4.3	6	42.5	na	5	57.1	2.3	56.5	3	36
**58**	4	8	na	3.4	13.6	na	3.9	15.1	na	na	10.5	50	10	49	28.5	47.5
**66**	3.8	12.5	54	na	na	na	4.7	2.7	47	na	16	39	na	na	2	54
**67**	na	na	na	na	na	na	5.7	11.7	45	16	na	na	na	na	25	47
**27**	4.8	6.5	51	na	na	na	3.6	8.9	45	N	4	48	6	57	7	43
**21**	6.1	4.6	55	3.1	8.1	62	5.2	8.6	43	19.9	32	43	na	na	8	45
**75**	3.8	5.1	53	3.7	10.2	59	3.3	Absent	32	na	2	50	4	50	Absent	Absent
**73**	na	na	na	2.5	12	na	4.7	12	47	na	12	57	na	na	16.5	46.5
**69**	na	na	na	3.1	11.2	na	3.9	16.6	na	12.8	na	na	na	na	18	49
**23**	2.9	9.5	55	2.5	10.8	68	3.5	17.3	43	na	21	54	17	53	23	44
**33**	na	na	na	2.5	11.3	59	3.8	10.8	49	5.9	17	54	11	61	30	56
**59**	4.7	9.1	54	3	8	57	5.8	0.8	42	na	14	41	6	43	9	55
**6**	na	na	na	na	na	na	5.6	11.6	49		na	na	na	na	20	46
**36**	4.1	6.9	59	na	na	na	5.6	8.7	49	na	30	53	12	50	21	47
**56**	3.9	3	60	3.1	9.5	58	5	5.4	51	na	19.3	56	5.3	54	10.1	44
**22**	na	na	na	na	na	na	4.5	12.3	47	na	na	na	na	na	25	56
**60**	5.7	5.4	49	3.2	5.7	50	na	na	na	na	3	33	1	67	na	ma
**32**	na	na	na	na	na	na	3.1	16.6	54	20.3	na	na	na	na	na	na
**35**	2.5	8.9	na	na	na	na	3.4	6.6	na	na	na	na	na	na	na	na
**19**	na	na	na	na	na	na	6.6	0.4	47	na	na	na	na	na	11	54

Case #	MOTOR NCS										SENSORY NCS					
	Median nerve			Ulnar nerve			Tibial nerve			MEP	Median nerve		Ulnar nerve		Sural nerve	
	DL <4 ms	CMAP amp >8mV	MCV >50 m/s	DL <3.5 ms	CMAP amp >8 mV	MCV >51 m/s	DL <5.5 ms	CMAP amp >7.5 mV	MCV >40 m/s	LL <17 ms	SNAP amp >10mV	SCV >49 m/s	SNAP amp >13mV	SCV >52 m/s	SNAP amp >9mV	SCV >40 m/s
**7**	na	na	na	na	na	na	3.5	4.6	53	na	na	na	na	na	3.1	42
**38**	3.6	7.8	59	2.6	11.3	50	3.7	10.6	55	11.4	14	58	7	58	6	48
**57**	4	9.5	55	2.7	12.4	na	5.8	1.1	40	na	15	56.5	12	55.5	32	40
**41**	4.4	4.8	56	2.1	9.4	56	6.7	1.7	53	na	7	46	5	50	8	62
**45**	na	na	na	na	na	na	5.1	12.5	40	na	na	na	na	na	9	51
**40**	na	na	na	na	na	na	3.2	6.6	48	na	na	na	na	na	6	53
**9**	3.7	9	55	na	na	na	4.1	7	46	na	5	43	8	62	na	na
**42**	na	na	na	2.4	12.8	58	3.6	12.7	na	15	20	52	na	na	26	52
**80**	4.2	20	33.8	2.8	13	36	5.6	na	29.2	na	14	36.7	9	37	6	25.6
**83**	na	na	na	na	na	na	6.4	3	42	na	18	49	9	46	Not elicit	na
**87**	na	na	na	8.1	11.5	50	11.5	4.2	46	na	14	na	5	na	13	na
**114**	2.7	8	57	na	na	na	4	2.6	70	na	28	54	na	na	15	54

DL-distal latency, CMAP-compound muscle action potential, SNAP-sensory nerve action potential. Amp-amplitude, MCV-motor conduction velocity, SCV-sensory conduction velocity, na-not available, LL-lower limbs.

Supplementary Figure e1. MRI brain scans showing cysts in the posterior fossa (A, B, C), thin corpus callosum (B, E, F) and cerebellar atrophy (G, H, I).

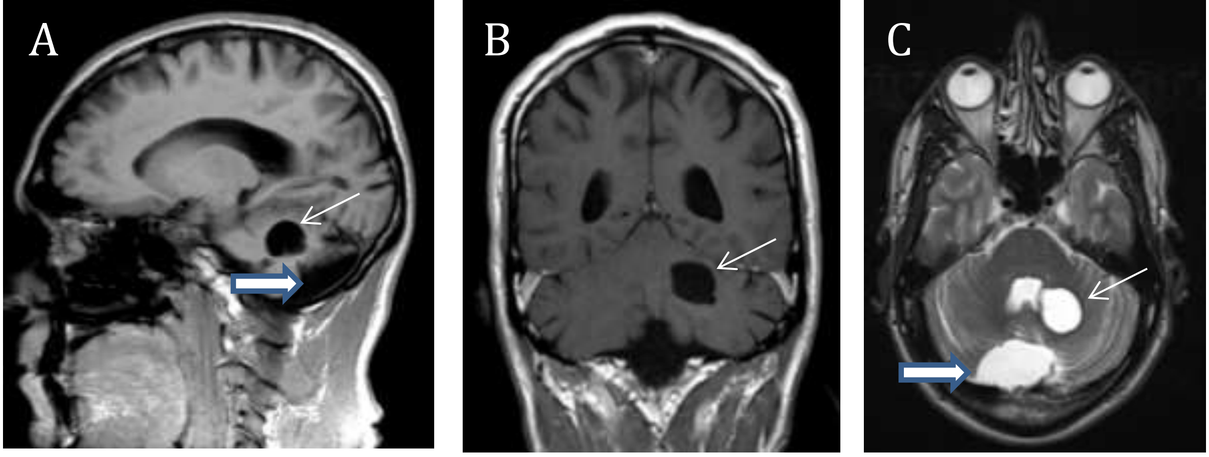

A-T1 sagittal view, B-T1 coronal view post gadolinium, C-T2 (case 69). The cystic lesion (narrow arrow) located in the left cerebellar lobe, indenting the lateral wall of the fourth ventricle presents a thin wall, with a few thin internal septa in its inferior portion. There is no evidence of enhancement of the wall after contrast administration, and no soft tissue components are identified. There is also a posterior fossa arachnoid cyst (large arrow).

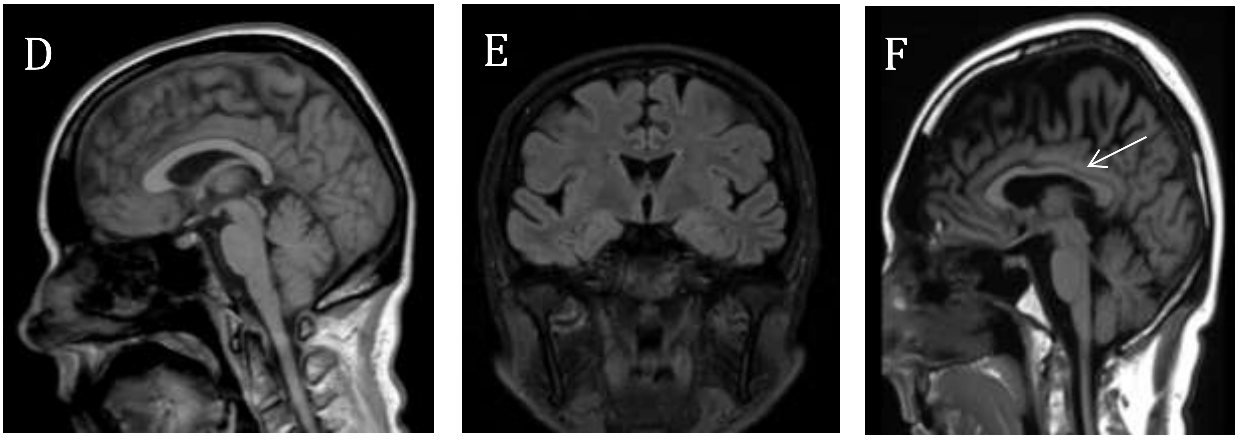

D and F-T1 sagital views-The splenium of the corpus callosum is mildely thinned and there is abnormal signal within it extending into the peritrigonal white matter (D, case 5) and moderatelly slender in the posterior 2/3rds (case 27). E- Few tiny nonspecific foci of abnormal signal elsewhere within the cerebral white matter.

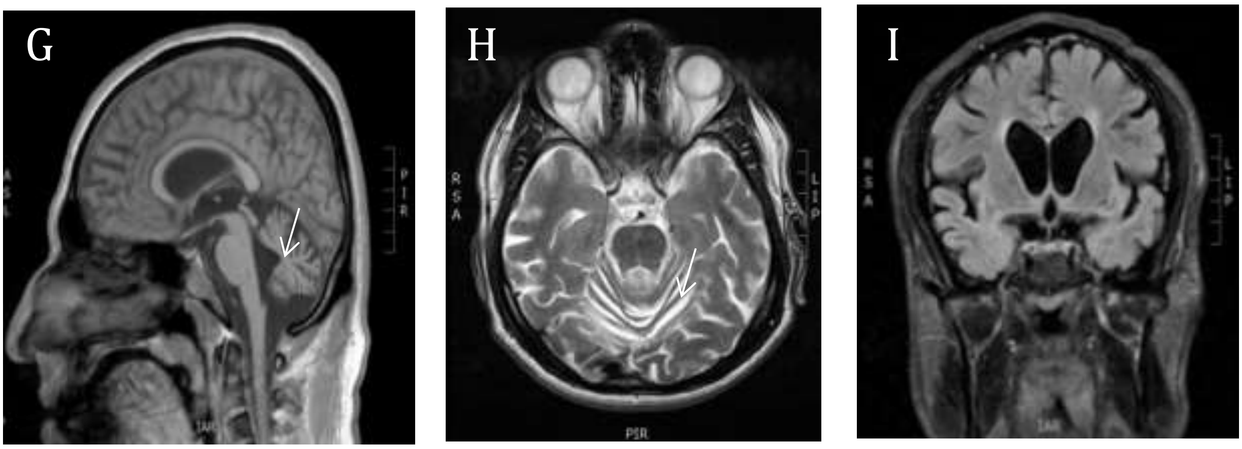

G-T1 sagittal view, H-T2, I-T2 coronal view (case 57). There is marked cerebellar volume loss (arrow) and supratentorial volume loss with minimal non-specific periventricular white matter abnormality.
Figure S1. MRI brain scans showing cysts in the posterior fossa (A, B, C), thin corpus callosum (B, E, F) and cerebellar atrophy (G, H, I).
