# Supplementary material for: Truncating mutations in SPAST patients are associated with a high rate of psychiatric comorbidities in hereditary spastic paraplegia
Source: J Neurol Neurosurg Psychiatry. 2017 Jun 1;88(8):681–7. doi: 10.1136/jnnp-2017-315796 (PMC5537546; doi:10.1136/jnnp-2017-315796)
Supplement: Supplementary Figure 1 [file jnnp-2017-315796supp002.pdf]

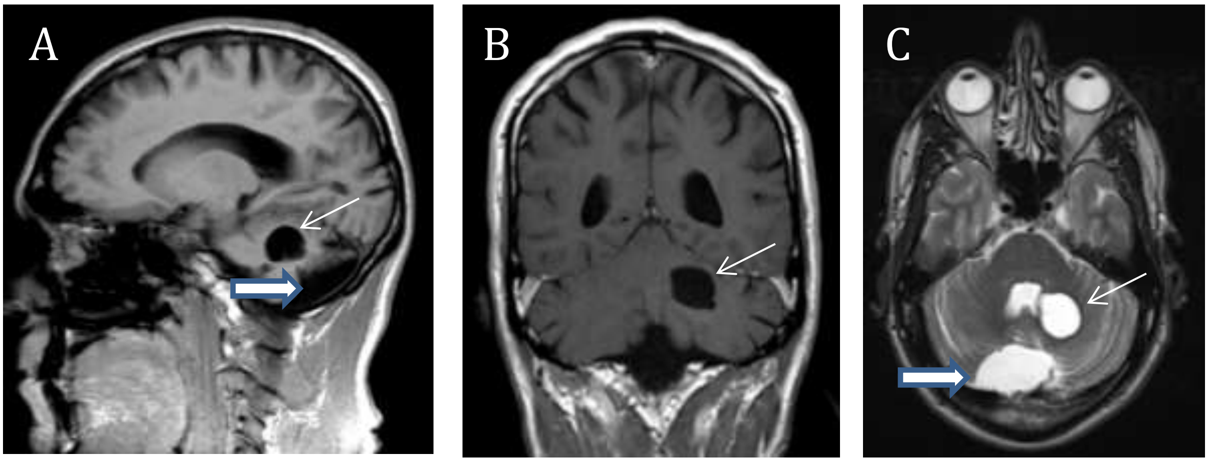

A-T1 sagittal view, B-T1 coronal view post gadolinium, C-T2 (case 69). The cystic lesion (narrow arrow) located in the left cerebellar lobe, indenting the lateral wall of the fourth ventricle presents a thin wall, with a few thin internal septa in its inferior portion. There is no evidence of enhancement of the wall after contrast administration, and no soft tissue components are identified. There is also a posterior fossa arachnoid cyst (large arrow).

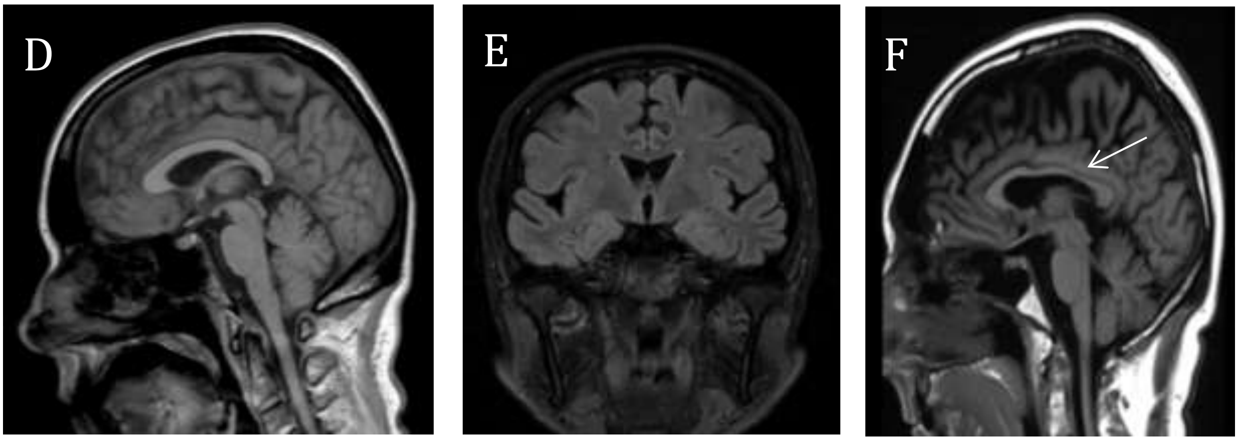

D and F-T1 sagital views-The splenium of the corpus callosum is mildely thinned and there is abnormal signal within it extending into the peritrigonal white matter (D, case 5) and moderatelly slender in the posterior 2/3rds (case 27). E- Few tiny nonspecific foci of abnormal signal elsewhere within the cerebral white matter.

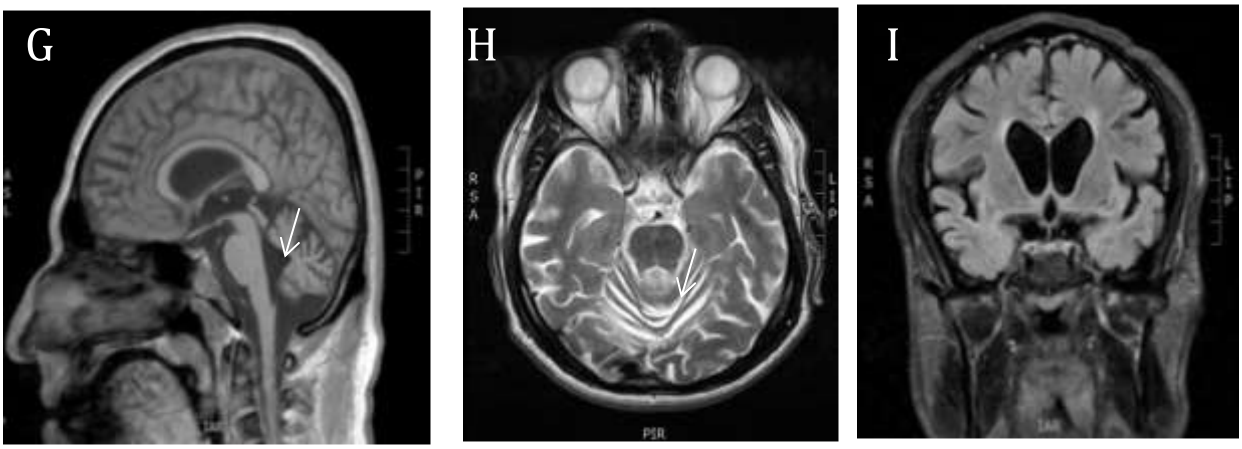

G-T1 sagittal view, H-T2, I-T2 coronal view (case 57). There is marked cerebellar volume loss (arrow) and supratentorial volume loss with minimal non-specific periventricular white matter abnormality.
Figure S1. MRI brain scans showing cysts in the posterior fossa (A, B, C), thin corpus callosum (B, E, F) and cerebellar atrophy (G, H, I).

Supplementary Figure e1. MRI brain scans showing cysts in the posterior fossa (A, B, C), thin corpus callosum (B, E, F) and cerebellar atrophy (G, H, I).
